# Synergistic inhibition of histone modifiers produces therapeutic effects in adult *Shank3*-deficient mice

**DOI:** 10.1038/s41398-021-01233-w

**Published:** 2021-02-04

**Authors:** Freddy Zhang, Benjamin Rein, Ping Zhong, Treefa Shwani, Megan Conrow-Graham, Zi-Jun Wang, Zhen Yan

**Affiliations:** Department of Physiology and Biophysics, State University of New York at Buffalo, Jacobs School of Medicine and Biomedical Sciences, Buffalo, NY 14203 USA

**Keywords:** Autism spectrum disorders, Epigenetics and behaviour

## Abstract

Autism spectrum disorder (ASD) is a lifelong developmental disorder characterized by social deficits and other behavioral abnormalities. Dysregulation of epigenetic processes, such as histone modifications and chromatin remodeling, have been implicated in ASD pathology, and provides a promising therapeutic target for ASD. Haploinsufficiency of the *SHANK3* gene is causally linked to ASD, so adult (3–5 months old) *Shank3*-deficient male mice were used in this drug discovery study. We found that combined administration of the class I histone deacetylase inhibitor Romidepsin and the histone demethylase LSD1 inhibitor GSK-LSD1 persistently ameliorated the autism-like social preference deficits, while each individual drug alone was largely ineffective. Another behavioral abnormality in adult *Shank3*-deficient male mice, heightened aggression, was also alleviated by administration of the dual drugs. Furthermore, Romidepsin/GSK-LSD1 treatment significantly increased transcriptional levels of NMDA receptor subunits in prefrontal cortex (PFC) of adult *Shank3*-deficient mice, resulting in elevated synaptic expression of NMDA receptors and the restoration of NMDAR synaptic function in PFC pyramidal neurons. These results have offered a novel pharmacological intervention strategy for ASD beyond early developmental periods.

## Introduction

Autism spectrum disorder (ASD) is a neurodevelopmental disorder characterized by impaired social communication, restricted interests and other behavioral abnormalities that last throughout the life span^1^. ASD affects approximately 1 in every 59 children in the United States, and is four times more prevalent in males than in females^2^. ASD is commonly diagnosed in early development, with the average age of diagnosis ranging from 3–10 years old^3,4^. Hence, the vast majority of preclinical studies in mouse models of ASD focuses on phenotypes in early development^5^. Presently there are no available pharmacotherapies for ASD-associated core deficits^6^.

ASD is strongly associated with *de novo* or inherited mutations in genes affecting convergent biological functions, including synaptic transmission and epigenetic control of transcription^7^. Haploinsufficiency of the *SHANK3* gene, which encodes a postsynaptic density scaffolding protein at glutamatergic synapses^8^, has been identified as a highly penetrant ASD risk factor^7,9,10^. Human patients carrying deletion of the 22q13.3 genetic region containing *SHANK3* (known as 22q13.3 deletion syndrome or Phelan-McDermid syndrome) exhibit ASD-associated phenotypes, including decreased socialization and intellectual disability^11^. Additionally, aggressive behavior is observed in 10–15% of patients with Phelan-McDermid syndrome^12^. In agreement with human phenotypes, young male mice with the heterozygous C-terminal deletion in the *Shank3* gene (Shank3^+/ΔC^) exhibit ASD-related social deficits^13,14^. Shank3^+/ΔC^ mice also display impaired N-methyl-D-aspartate (NMDA) receptor-mediated glutamatergic transmission in the prefrontal cortex (PFC)^13,14^, a brain region critical for high level executive function and social cognition^15,16^.

Epigenetic processes that are involved in histone modification and chromatin remodeling have been identified as commonly dysregulated pathways in ASD^7,17^. DNA packaging proteins known as histones can be post-translationally modified to induce transcriptional activation or repression^18^. Several of these modifications, such as histone acetylation or methylation, are dynamically regulated by epigenetic enzymes, including histone acetyltransferases (HAT) and deacetylases (HDAC), histone methyltransferases (HMT) and demethylases (HDM)^18^. Class I HDACs, which mediate gene repression through the removal of gene-activating histone acetylation, form complexes with various epigenetic enzymes to regulate diverse gene classes^19^. One such binding partner of class I HDAC is LSD1 (lysine-specific demethylase 1, KDM1A), a histone demethylase that enacts gene repression by removing histone 3 lysine 4 methylation that is linked to gene activation^20^. Class I HDAC and LSD1 are often recruited to the same chromatin-associated multi-protein complex to regulate the transcription of common genes^21,22^. Several recent studies have demonstrated that compounds targeting epigenetic enzymes, such as class I HDAC or EHMT1/2, are capable of restoring sociability in young (juvenile to adolescent) Shank3^+/ΔC^ mice^13,23,24^. While this represents a promising therapeutic strategy in ASD, its applicability in adulthood is largely unknown.

In the current study, we report that robust and prolonged amelioration of behavioral deficits in adult (3–5 months old) Shank3^+/ΔC^ mice is achieved via the combined administration of a class I HDAC inhibitor and an LSD1 inhibitor. When dually administered, these compounds restore NMDAR synaptic expression and function in PFC. These findings suggest that synergistic inhibition of class I HDAC and LSD1 may represent an effective treatment strategy for adults with ASD.

## Materials and methods

### Animals and drugs

Mice expressing C-terminal (exon 21)-deleted Shank3 (Shank3^+/ΔC^) were obtained from Jackson Labs (Bar Harbor, ME) in early 2013 (stock#: 018398). They were backcrossed for over 10 generations onto the C57BL/6 inbred strain and were maintained on the C57BL/6 genetic background, as previously described^13,14^. Adult heterozygous Shank3^+/ΔC^ mice (3–5 months old, male) and age-matched wild-type littermates (male) were used in the current study. Mice were group-housed with ad libitum food accessibility in a 12-hr light–dark cycle (light: 6 a.m.–6 p.m.; dark: 6 p.m.–6 a.m.). Mice of each genotype were randomly assigned to drug/saline groups. Experiments were carried out by investigators with no prior knowledge of genotypes or treatments. All experiments were performed with the approval of the Institutional Animal Care and Use Committee of the State University of New York at Buffalo.

Romidepsin (Selleck Chem) and GSK-LSD1 dihydrochloride (Tocris) were dissolved into DMSO, then diluted with 0.9% saline before injection. Romidepsin (0.25 mg/kg) and GSK-LSD1 (5 mg/kg) were administered separately, via intraperitoneal injection. Each drug was administered three times: once per day over the course of three subsequent days. All behavioral tests were performed at various time points after the final injection of the compound (as indicated in the paper). All biochemical experiments were performed with prefrontal cortical tissue collected 8 days after the final treatment, unless otherwise indicated.

### Behavioral testing

#### Three-chamber social preference

Three-chamber social interaction assay was performed to assess social deficits as previously described^13,14^. For habituation, the test mouse was placed into a Plexiglass arena (L: 101.6 cm, W: 50.8 cm, H: 50.8 cm) containing two empty inverted pencil cups (D: 10.2 cm, H: 10.5 cm) in two side chambers for 10 min. An upright glass water bottle was placed on top of each pencil cup to prevent the test mouse from climbing the cup. On the following day, the mouse was reintroduced to the apparatus for a 10-min pre-test trial, in which the pencil cups contained two identical objects (paper balls). The animal was then returned to its home cage for 5 min. The animal was then placed into the apparatus for a 10-min social trial, in which one cup contained a non-social stimulus (NS; wooden block) and the other contained a social stimulus (Soc; an age- and gender-matched unfamiliar WT mouse). Locations of the NS and Soc stimuli were counterbalanced between animals, and the apparatus was cleaned between trials. The amount of time spent interacting with each stimulus over the 10-min period was recorded. For repeated tests, a new stimulus mouse was used at each assay to avoid habituation to the social stimulus. Interaction time was counted based on ‘investigating’ behaviors of the test animal toward each stimulus, and was scored manually. The preference index was calculated as [(social time – non-social time)/(social time + non-social time)] × 100.

#### Locomotion

The total distance traveled during the test phase of the three-chamber social preference test was measured by a computer running ANY-maze behavior tracking software (Stoelting, Wood Dale, IL).

#### Resident intruder (RI) Test

Animals were subject to the RI test as previously described^25,26^. Briefly, each mouse was single housed for 24 h prior to the RI test. The resident mouse (male) was then exposed to an intruder, which was a smaller (~50% lighter) unfamiliar male WT mouse, in its home cage for 10 min. The total amount of time that the resident mouse spent attacking the intruder mouse (defined as chasing, prodding, or fighting) was manually scored to measure aggression-related behaviors. The intruders were all socially experienced, but naïve to the RI test. Each intruder was used only once and was not re-used for other aggressive encounters (to avoid winner or loser effects).

### Quantitative polymerase chain reaction (qPCR)

Total RNA was isolated from mouse PFC punch biopsy samples using Trizol reagent (Invitrogen) and treated with DNase I (Invitrogen) to remove genomic DNA from the sample. Then SuperScript III first-strand synthesis system (Invitrogen) was used to obtain cDNA from the tissue mRNA, followed by the treatment with RNase H (2 U/ l) for 20 min at 37 °C. Quantitative PCR was carried out using the iCycler iQ Real-Time PCR Detection System and iQ Supermix (Bio-Rad) according to the manufacturer’s instructions. A total reaction mixture of 25 μL was amplified in a 96-well thin-wall PCR plate (Bio-Rad) using the following PCR cycling parameters: 95 °C for 5 min, followed by 40 cycles of 95 °C for 30 s, 55 °C for 30 s and 72 °C for 60 s. *Gapdh* was used as the housekeeping gene for quantitation. Fold changes in the target genes were determined by: Fold change = 2^-Δ(ΔC^_T_^)^, where ΔC_T_ = C_T(target)_ – C_T(GAPDH)_, and Δ(ΔC_T_) = ΔC_T(treated group)_ - ΔC_T(WT+saline)_. Primers for all the genes profiled in this study are:

**Table:** 

Gene	Forward	Reverse
***Gapdh***	GACAACTCACTCAAGATTGTCAG	ATGGCATGGACTGTGGTCATGAG
***Grin1***	CATCGGACTTCAGCTAATCA	GTCCCCATCCTCATTGAATT
***Grin2a***	GGCTACAGAGACTTCATCAG	ATCCAGAAGAAATCGTAGCC
***Grin2b***	TTAACAACTCCGTACCTGTG	TGGAACTTCTTGTCACTCAG
***GluR1***	GCCTTAATCGAGTTCTGCTA	GAATGGATTGCATGGACTTG
***GluR2***	AGCCTATGAGATCTGGATGT	GAGAGAGATCTTGGCGAAAT
***Shank3***	GATCTGCCATCCCTACAAC	AGCTAAGGGTGAGCTAGGAT
***Homer1b***	AACACAAAGAAGAACTGGGT	ATTGCCTTTGAGCCATCTAA
***Arc***	GAGGCTCAGCAATATCAGTC	GGACAGCCAATATTCTTCAG

### Biochemical measurement of synaptic proteins

Synaptic membrane protein fraction was prepared as described previously^14^. In brief, blocks of frontal cortex were dissected, weighed and homogenized in ice-cold lysis buffer (10 mL/g, 15 mM Tris, pH 7.6, 0.25 M sucrose, 1 mM PMSF, 2 mM EDTA, 1 mM EGTA, 10 mM Na_3_VO_4_, 25 mM NaF, 10 mM sodium pyrophosphate and protease inhibitor tablet). After centrifugation at 800 x g for 5 min to remove nuclei and large debris, the remaining supernatant was subjected to 10,000 x g centrifugation for 10 min. The crude synaptosome fraction (pellet) was suspended in lysis buffer containing 1% Triton X-100 and 300 mM NaCl, homogenized again and centrifuged at 16,000 x g for 15 min. The Triton insoluble fraction, which mainly includes membrane-associated proteins from synaptosomes, was dissolved in 1% SDS. Samples were boiled in 2 × SDS loading buffer for 5 min and separated on 7.5% SDS-PAGE. Western blots were performed using antibodies against NR1 (1:500, NeuroMab, 75‐ 272), NR2A (1:500, Millipore, 07‐632), NR2B (1:500, Millipore, 06‐600), PSD95 (1:1000, Cell Signaling, 36233 S) and tubulin (1:5000 Sigma, T9026).

### Immunohistochemistry

Mice were anesthetized and transcardially perfused with PBS, followed by 4% paraformaldehyde (PFA) before brain removal. Brains were post-fixed in 4% PFA for 1 day prior to 3-day incubation in 30% sucrose, then cut into 50-μm coronal slices. Prior to immunohistochemistry, slices were washed and blocked for 1 h in PBS containing 5% donkey serum and 0.3% Triton for permeabilization. After washing, slices were incubated with the primary antibody against H3K9ac (1:500, Cell Signaling Technology, 9649 S) for 24 h at 4 °C. After washing three times (15 min with gentle shaking) in PBS, slices were incubated with secondary antibody (Alexa Fluor 568, A10042 or Alexa Fluor 488, SA5-1009, Invitrogen, both 1:400) for 24 h at 4 °C, followed by three washes with PBS. Slices were mounted on slides with Vectashield mounting media with DAPI (Vector Laboratories). Images were acquired using a Leica DMi8 confocal fluorescence microscope. All specimens were imaged under identical exposure conditions and analyzed with identical parameters using ImageJ software.

### Electrophysiology

Whole-cell voltage-clamp recording technique was used to measure synaptic currents in layer V pyramidal neurons of prefrontal cortical slices as previously described^13^. Mouse slices (300 μm) were positioned in a perfusion chamber attached to the fixed stage of an upright microscope (Olympus) and submerged in continuously flowing oxygenated artificial cerebrospinal fluid (in mM: 130 NaCl, 26 NaHCO3, 1 CaCl_2_, 5 MgCl_2_, 3 KCl, 1.25 NaH_2_PO_4_, 10 glucose, pH 7.4, 300 mOsm). The GABA antagonist bicuculline (20 μM) and AMPA antagonist CNQX (20 μM) were added in NMDAR-EPSC recordings. Bicuculline and D-APV (50 μM) were added in AMPAR-EPSC recordings. Patch electrodes contained internal solution (in mM): 130 Cs-methanesulfonate, 10 CsCl, 4 NaCl, 10 HEPES, 1 MgCl_2_, 5 EGTA, 2 QX-314, 12 phosphocreatine, 5 MgATP, 0.2 Na_3_GTP, 0.1 leupeptin, pH 7.2–7.3, 265–270 mOsm. Neurons were visualized with a 40x water-immersion lens and recorded with the Multiclamp 700 A amplifier (Molecular Devices, Sunnyvale, CA). Evoked EPSC were generated with a pulse from a stimulation isolation unit controlled by a S48 pulse generator (Grass Technologies, West Warwick, RI). A bipolar stimulating electrode (FHC, Bowdoinham, ME) was placed ~100 μm from the neuron under recording. For NMDAR-EPSC, the cell (clamped at −70 mV) was depolarized to +40 mV for 3 s before stimulation to fully relieve the voltage-dependent Mg^2+^ block. For AMPAR-EPSC, the membrane potential was maintained at −70 mV. For input–output responses, EPSC was elicited by a series of pulses with different stimulation intensities (50–90 μA) delivered at 0.033 Hz. To obtain NMDAR-to-AMPAR-EPSC ratio, AMPAR-EPSC was first recorded at −70 mV in ACSF solution (containing bicuculline). Then the mixture of AMPAR-EPSC and NMDAR-EPSC was recorded at +40 mV with the same stimulation pulse (0.4 ms, 80 µA). The peak of NMDAR-EPSC was calculated at 40 ms from the onset of the EPSC mixture. Spontaneous EPSCs (sEPSC) were recorded with the same external and internal solutions in neurons clamped at −70 mV. Data analyses were performed with Clampfit (Axon instruments, Molecular Devices, Sunnyvale, CA), Mini Analysis Program 6.0.3 (Synaptosoft), Kaleidagraph (Albeck Software, Synergy Software, Reading, PA) and GraphPad Prism 6 (GraphPad Software, Inc., La Jolla, CA).

### Statistics

All data are presented as mean ± SEM. No sample was excluded from the analysis. The sample size was based on power analyses and was similar to those reported in previous work^14,27,28^. The variance between groups being statistically compared was similar. Each set of the experiments was replicated in at least three independent groups of animals. Data-points identified as statistically significant outliers were removed from the analyses. Outliers were determined via Grubb’s test as data-points falling above a critical Z-score (determined by sample size) where *p* < 0.05. Experiments with two groups were analyzed statistically using unpaired Student’s *t*-tests with Welch’s correction. Experiments with more than two groups were subjected to one-way ANOVA, two-way ANOVA, or two-way repeated measure ANOVA (rmANOVA), followed by post hoc comparisons with Bonferroni corrections for multiple comparisons.

## Results

### Combined administration of HDAC inhibitor Romidepsin and LSD1 inhibitor GSK-LSD1 exerts persistent rescue of social deficits in adult Shank3-deficient mice

**Fig. 1:**
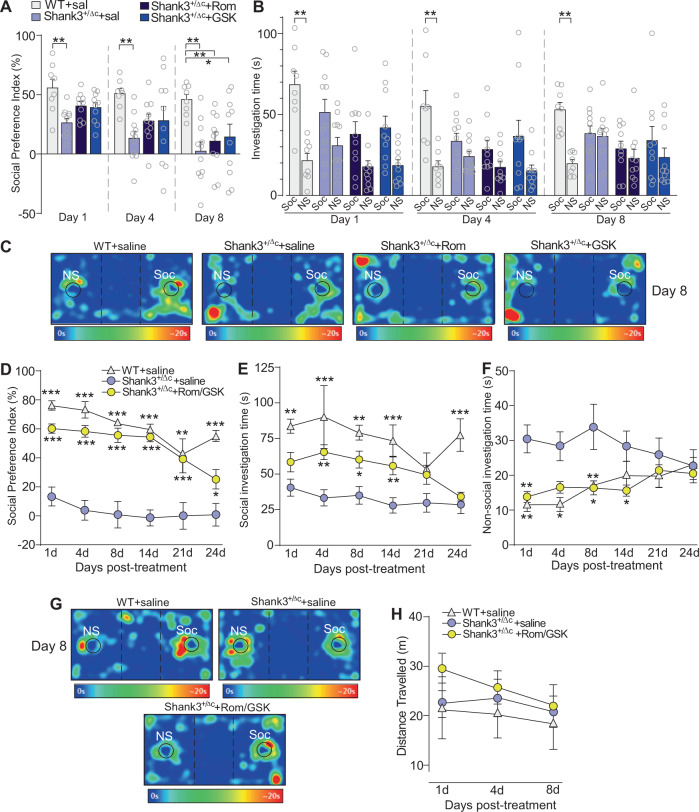
Combined administration of Romidepsin and GSK-LSD1 exerts persistent rescue of social deficits in adult male Shank3-deficient mice. **A** Bar graphs showing social preference index of adult saline-treated WT or Shank3^+/ΔC^ mice treated with saline, Romidepsin (Rom, 0.25 mg/kg, i.p., 3x) or GSK-LSD1 (5 mg/kg, i.p., 3x) at 1, 4, and 8 days post-treatment in the 3-chamber social preference test. **B** Bar graphs showing social (Soc) and non-social (NS) investigation time of the four groups at 1, 4, and 8 days post-treatment. For panels **A** & **B**, WT + sal: *n* = 8 mice; Shank3+sal: *n* = 11 mice; Shank3+Rom: *n* = 10 mice; Shank3+GSK: *n* = 10 mice. **C** Representative heat maps for all four groups at day 8 post-treatment. **D** Plots of social preference index for adult saline-treated WT or Shank3^+/ΔC^ mice treated with saline or both Romidepsin (0.25 mg/kg, i.p., 3x) and GSK-LSD1 (5 mg/kg, i.p., 3x) (Rom/GSK) at days 1–24 post-treatment in the 3-chamber social preference test. **E, F** Plot of social (**E**) and non-social (**F**) investigation time of the three groups at days 1–24 post-treatment. For panels D-F, WT + sal; *n* = 7 mice; Shank3+sal: *n* = 15 mice; Shank3+Rom/GSK: *n* = 12 mice. **G** Representative heat maps for all three groups at day 8 post-treatment. **H** Plot showing distance traveled during the test phase of the three-chamber social preference assay for the 3 groups at various time points. WT + sal: *n* = 6 mice; Shank3+sal: *n* = 15 mice; Shank3+Rom/GSK: *n* = 6 mice. In all figures, *p < 0.05, **p < 0.01, ***p < 0.001. In panels **D**–**F**, asterisks indicate significant differences when compared with Shank3^+/ΔC^ + Saline group within a given time point.

Pharmacological targeting of histone modifiers has been established as an effective method for rescuing ASD-related social deficits in adolescent (5–8 weeks old) Shank3-deficient (Shank3^+/ΔC^) mice^13,23,24^. However, it remains unclear whether these therapeutic effects can be similarly obtained in adulthood. Therefore, we treated adult (3–5 months old) male Shank3^+/ΔC^ mice with either the highly potent and brain permeable^29^ class I HDAC inhibitor Romidepsin^30^ (0.25 mg/kg, i.p. once daily for 3 days), or the selective LSD1 inhibitor GSK-LSD1^31^ (5 mg/kg, i.p., daily for 3 days), and assessed their sociability with the three-chamber social preference test. Compared to saline-treated adult wild-type (WT) mice, saline-treated adult Shank3^+/ΔC^ mice displayed a significantly lower social preference index at all time points tested, confirming the presence of social deficits in Shank3^+/ΔC^ mice that persists into adulthood (Fig. 1A). Adult Shank3^+/ΔC^ mice treated with Romidepsin or GSK-LSD1 alone exhibited a slight improvement of social preference index at day 1 or day 4 post-treatment (Fig. 1A, *n* = 8–11 mice/group, *F*_2,70 (time)_ = 10.1, *p* = 0.0001; *F*_3,35 (group)_ = 11.5, *p* < 0.0001, two-way rmANOVA). No significant improvement was observed on the social interaction time with either drug treatment at all tested time points (Fig. 1B, day 1: *F*_3,70 (group)_ = 3.3, *p* = 0.03; day 4: *F*_3,70 (group)_ = 1.7, *p* = 0.17; day 8: *F*_3,70 (group)_ = 2.1, *p* = 0.11, two-way ANOVAs). Representative heat maps illustrating the time spent in different locations of the 3-chamber apparatus for all groups at day 8 post-treatment are shown in Fig. 1C. These data indicate that targeting each individual epigenetic enzyme only exerts transient and modest effects in adult Shank3^+/ΔC^ mice.

Since class I HDACs function cooperatively with LSD1 in repressor complexes^19,21,22^, we next co-administered Romidepsin and GSK-LSD1 to assess whether combined inhibition of HDACs and LSD1 may have synergistic effects to rescue sociability in adult Shank3-deficient mice. Remarkably, adult Shank3^+/ΔC^ mice treated with both Romidepsin and GSK-LSD1 (0.25 mg/kg and 5 mg/kg, respectively, i.p., 3 days) displayed significantly elevated social preference indexes relative to saline-treated adult Shank3^+/ΔC^ mice, which persisted for 24 days post-treatment (Fig. 1D, *n* = 7–15 mice/group, *F*_2,31 (group)_ = 53.4, *p* < 0.0001, two-way rmANOVA). Combined treatment of adult Shank3^+/ΔC^ mice with romidepsin and GSK-LSD1 also significantly increased the time interacting with social stimuli (Fig. 1E, *F*_2,31 (group)_ = 16.66, *p* < 0.0001, two-way rmANOVA), and significantly decreased the time interacting with non-social stimuli (Fig. 1F, *F*_2,31 (group)_ = 8.0, *p* = 0.002, two-way rmANOVA). Representative heat maps illustrating the time spent in different locations of the three-chamber apparatus for all groups at day 8 post-treatment are shown in Fig. 1G. Collectively, these data suggest that the combined inhibition of HDAC and LSD1 produces potent and long-lasting rescue of social deficits in adult Shank3^+/ΔC^ mice.

To ensure that the observed increases in sociability were not caused by drug-induced changes in locomotion, we assessed locomotive activity in Rom/GSK-treated Shank3^+/ΔC^ mice. No significant differences in locomotion were observed during the test phase of the 3-chamber social preference assay among WT or Shank3^+/ΔC^ mice treated with saline or Rom/GSK (Fig. 1H, *n* = 6–15 mice/group, *F*_2,24 (group)_ = 0.5, *p* = 0.6, two-way rmANOVA).

### Combined administration of Romidepsin and GSK-LSD1 ameliorates heightened aggression in adult Shank3-deficient mice

**Fig. 2:**
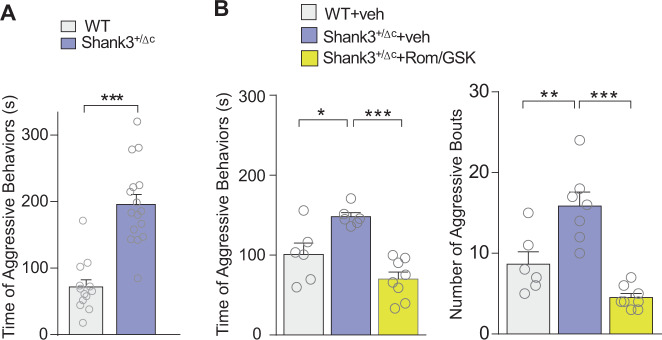
Treatment with Romidepsin and GSK-LSD1 ameliorates hyper-aggressive behavior in adult male Shank3^+/ΔC^ mice. **A** Plot showing the time of aggressive behaviors in the resident-intruder (RI) test of adult male WT mice (*n* = 13) and Shank3^+/ΔC^ mice (*n* = 16). **B** Plot showing the time of aggressive behaviors and the number of aggressive bouts in the RI test of adult male WT or Shank3^+/ΔC^ mice treated with Rom/GSK (0.25/5 mg/kg, i.p., 3x) or vehicle control. *n* = 7–8 mice per group. In all figures, *p < 0.05, **p < 0.01, ***p < 0.001.

As Shank3^+/ΔC^ male mice grow into adulthood, we observed that they often sustained injuries from fighting with cage-mates. We thus assessed their aggressive behavior using the resident-intruder (RI) test. Compared to adult WT controls, adult Shank3^+/ΔC^ mice spent significantly more time engaging in aggressive behaviors with the intruder mouse (Fig. 2A, WT: *n* = 13, Shank3^+/ΔC^: *n* = 16, *t*_(26)_ = 6.7, *p* < 0.001, *t*-test). However, adult Shank3^+/ΔC^ mice treated with Romidepsin and GSK-LSD1 had significantly decreased aggression time (Fig. 2B, *n* = 6–8 mice/group, *F*_2,17_ = 16.06, *p* = 0.0001, one-way ANOVA) and significantly fewer bouts of aggressive behaviors (Fig. 2C, *n* = 7–8 mice/group, *F*_2,18_ = 21.05, *p* < 0.0001, one-way ANOVA), compared to Shank3^+/ΔC^ mice with vehicle treatment, which were similar to WT mice treated with vehicle. Altogether, these data have revealed a new phenotype in adult *Shank3*-deficient mice – heightened aggression, which is ameliorated by combined inhibition of HDAC and LSD1.

### Combined administration of Romidepsin and GSK-LSD1 increases NMDAR transcription and restores NMDAR function in adult Shank3^+/ΔC^ mice

**Fig. 3:**
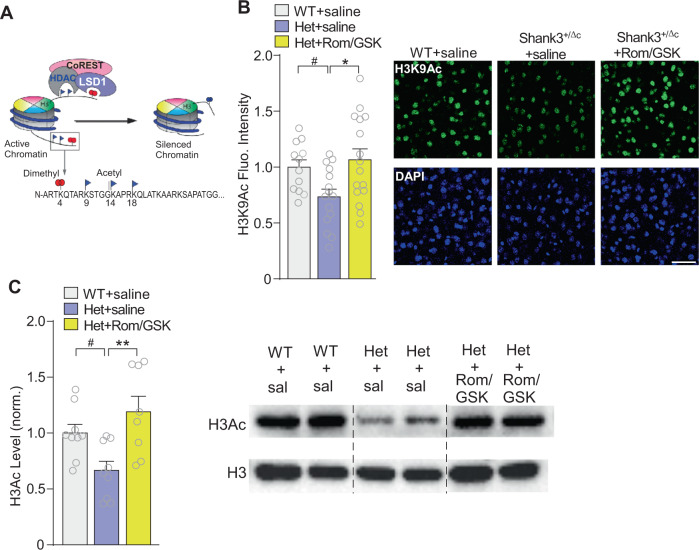
Treatment with Romidepsin and GSK-LSD1 elevates histone acetylation in PFC of adult Shank3^+/ΔC^ mice. **A** Diagram showing chromatin regulation by the concerted action of histone modifiers. LSD1 (removing permissive H3K4me2, red circles) is a key partner of HDAC (removing permissive H3Ac, navy blue triangles) in the CoREST repressor complex, which collectively contributes to chromatin remodeling and repression of downstream genes. **B** Quantification of immunofluorescence intensity of histone 3 lysine 9 acetylation (H3K9ac) in medial PFC of adult saline-treated WT or Shank3^+/ΔC^ mice treated with saline or Rom/GSK (0.25/5 mg/kg, i.p., 3x) at 8 days post-treatment. *n* = 12–16 slices/3 mice per group. Inset: Representative immunohistochemistry images. Scale bar = 50 μm. **C** Quantification of acetylated-histone 3 (H3) in medial PFC of adult saline-treated WT or Shank3^+/ΔC^ mice treated with saline or Rom/GSK (0.25/5 mg/kg, i.p., 3x) at 8 days post-treatment. Values normalized to total H3. Inset: Representative western blotting images. In all figures, ^#^*p* < 0.1, *p < 0.05, **p < 0.01.

Next, we sought to find out the mechanism underlying behavioral rescue by dual inhibitors. Since class I HDAC and LSD1 are often recruited to the same chromatin-associated complex to regulate the transcription of common genes^21,22^, we hypothesize that the synergistic effect of Rom/GSK is driven by the combined inhibition of two functionally coupled epigenetic enzymes (Fig. 3A). For these biochemical studies, we focused on the prefrontal cortex (PFC), as this brain region is highly implicated in social cognition^15,16,32,33^. We first examined the impact of dual inhibitors on histone acetylation in PFC neurons. Compared to saline-treated WT mice, adult Shank3^+/ΔC^ mice had significantly reduced level of histone 3 lysine 9 acetylation (H3K9ac), which was reversed by combined treatment with Romidepsin and GSK-LSD1 (Fig. 3B, *n* = 12–16 slices/3 mice/group, *F*_2,40_ = 5.0, *p* = 0.01, one-way ANOVA). Restoration of histone 3 acetylation (H3Ac) was further verified via Western blotting, which similarly indicated the rescue of H3Ac in PFC of Rom/GSK-treated Shank3^+/ΔC^ mice (Fig. 3C, *n* = 8–10 mice, *F*_2,23_ = 7.09, *p* = 0.004, one-way ANOVA). These results indicate that Rom/GSK treatment may alleviate social deficits by reversing transcriptional changes driven by aberrant histone modifications.

**Fig. 4:**
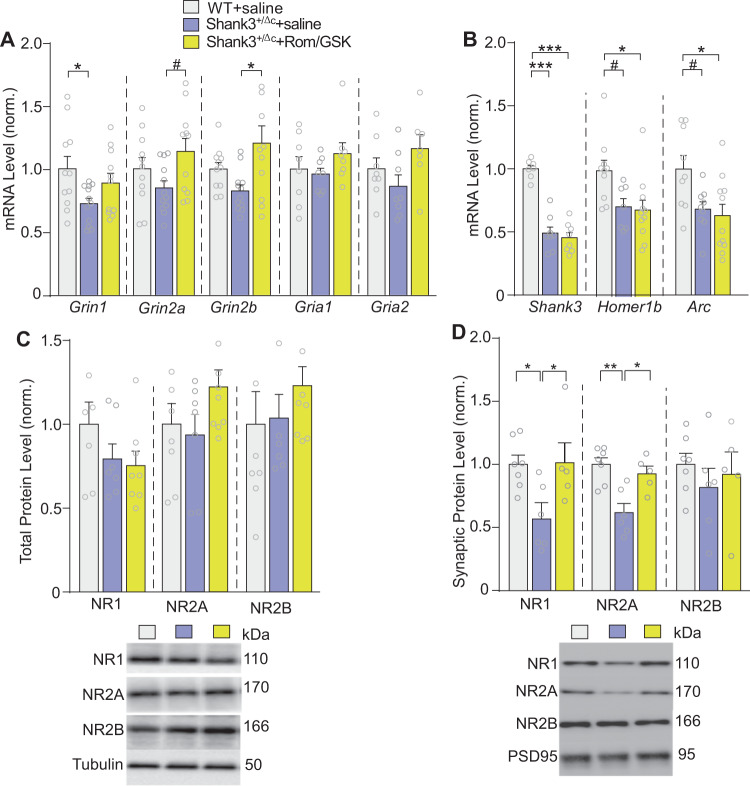
Treatment with Romidepsin and GSK-LSD1 induces transcriptional upregulation of NMDAR subunits and restores synaptic NMDAR expression in PFC of adult Shank3^+/ΔC^ mice. **A**, **B** Bar graphs showing mRNA levels of NMDAR subunits (*Grin1, Grin2a, Grin2b*) and AMPAR (*Gria1, Gria2*) subunits (**A**) and synaptic proteins (*Shank3*, *Homber1b*, *Arc*) (**B**) in PFC of adult saline-treated WT or Shank3^+/ΔC^ mice treated with saline or Rom/GSK (0.25/5 mg/kg, i.p., 3x) at 8 days post-treatment. *n* = 7–11 mice per group. **C, D** Bar graphs showing total (**C**) or synaptic (**D**) protein levels of NMDAR subunits in the 3 groups at 8 days post-treatment. C, *n* = 7–9 mice/group; D, *n* = 5–7 mice per group. Insets: representative immunoblots. In all figures, ^#^*p* < 0.1, *p < 0.05, **p < 0.01, ***p < 0.001.

Increased H3K9ac by Rom/GSK treatment could lead to the upregulation of gene transcription. Next, we performed qPCR experiments to identify altered genes in PFC of adult Shank3^+/ΔC^ mice treated with Romidepsin and GSK-LSD1. Since young Shank3^+/ΔC^ mice exhibit deficient NMDAR-mediated synaptic transmission in PFC^14^, we first examined the mRNA level of NMDAR subunits. As shown in Fig. 4A, NR2A (*Grin2a*) and NR2B (*Grin2b*) subunits were significantly increased in PFC of Rom/GSK-treated Shank3^+/ΔC^ mice, compared to saline controls (*n* = 11–12 mice/group, *Grin2a:* *F*_2,31_ = 3.1, *p* = 0.06; *Grin2b*: *F*_2,31_ = 4.8, *p* = 0.015; one-way ANOVA). NR1 (*Grin1*), which was significantly decreased in adult Shank3^+/ΔC^ mice, was also elevated by Romidepsin and GSK-LSD1 treatment (*F*_2,31_ = 3.4, *p* = 0.05, one-way ANOVA). The mRNA level of AMPA receptor GluR1 (*Gria1*) and GluR2 (*Gria2*) subunits were largely unchanged in PFC of Rom/GSK-treated Shank3^+/ΔC^ mice (Fig. 4A, *n* = 7–8 mice/group, *Gria1*: *F*_2,21_ = 1.1, *p* = 0.4; *Gria2*: *F*_2,20_ = 2.4, *p* = 0.1; one-way ANOVA). These findings indicate that combined inhibition of HDAC and LSD1 induces transcriptional upregulation of NMDARs in adult Shank3^+/ΔC^ mice.

We further examined the effect of dual inhibitors on additional genes involved in synaptic plasticity. Other than *Shank3*, we profiled the mRNA level of *Homer1b*, the mGluR scaffolding protein that is altered in *Shank3* complete knockout mice^34^, and *Arc*, an activity-associated immediate early gene highly involved in schizophrenia, intellectual disability and autism^35^. As shown in Fig. 4B, these genes were significantly downregulated in PFC of adult Shank3^+/ΔC^ mice, consistent with our finding in young Shank3^+/ΔC^ mice^13,23,24^, but combined treatment with Romidepsin and GSK-LSD1 failed to elevate them (*Shank3*: *n* = 7–8 mice/group, *F*_2,20_ = 51.6, *p* < 0.0001; *Homer1b*: *n* = 7–11 mice/group, *F*_2,25_ = 5.1, *p* = 0.01; *Arc*: *n* = 9–11 mice/group, *F*_2,26_ = 5.1, *p* = 0.01; one-way ANOVA).

Given the transcriptional upregulation of NMDAR subunits by dual inhibitors, we further examined NMDAR protein levels. No significant changes in NR1, NR2A, or NR2B were observed in total lysates, despite a trend of increase on NR2A and NR2B in Rom/GSK-treated Shank3^+/ΔC^ mice (Fig. 4C, *n* = 7–9 mice/group, NR1: *F*_2,20_ = 0.7, *p* = 0.7; NR2A: *F*_2,21_ = 1.7, *p* = 0.2; NR2B: *F*_2,21_ = 0.7, *p* = 0.5; one-way ANOVAs). In fractions containing only membrane-associated synaptic proteins isolated from PFC tissue, we found a significant loss of synaptic NR1 and NR2A in saline-treated Shank3^+/ΔC^ mice relative to WT, and the synaptic level of NR1 and NR2A, but not NR2B, was significantly elevated in Rom/GSK-treated Shank3^+/ΔC^ mice (Fig. 4D, *n* = 5–7 mice/group, NR1: *F*_2,15_ = 5.2, *p* = 0.02; NR2A: *F*_2,15_ = 10.8, *p* = 0.001; NR2B: *F*_2,15_ = 0.5, *p* = 0.6; one-way ANOVA).

**Fig. 5:**
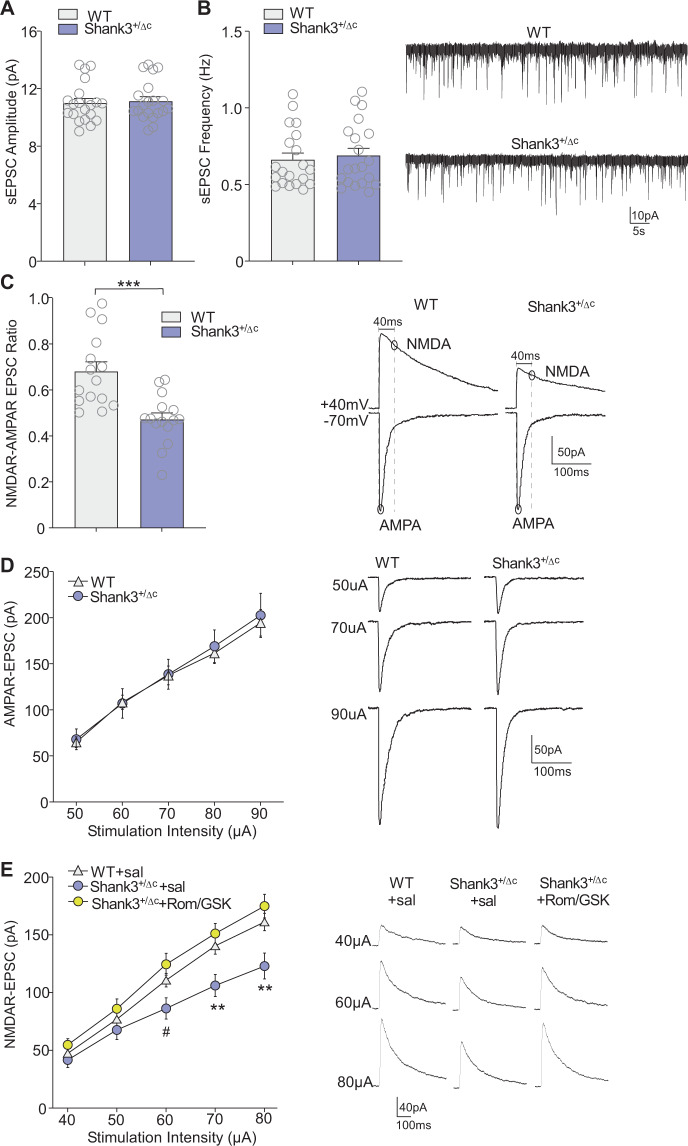
Treatment with Romidepsin and GSK-LSD1 restores NMDAR function in PFC pyramidal neurons of adult Shank3^+/ΔC^ mice. **A**, **B** Bar graphs (mean ± SEM) of sEPSC amplitude (**A**) and frequency (**B**) in PFC pyramidal neurons from WT vs. Shank3^+/ΔC^ mice (*n* = 20 cells/3–4 mice per group). Inset: representative sEPSC traces. **C** Bar graphs (mean ± SEM) of NMDAR-EPSC-to-AMPAR-EPSC ratio in PFC pyramidal neurons from WT and Shank3^+/ΔC^ mice (*n* = 15 cells/3–4 mice per group). Inset: representative traces. **D** Input-output curves of AMPAR-EPSC in PFC pyramidal neurons from WT vs. Shank3^+/ΔC^ mice (*n* = 13–15 cells from 3–4 mice per group). Inset: representative AMPAR-EPSC traces. **E** Inputoutput curves of NMDAR-EPSC in PFC pyramidal neurons from adult saline-treated WT or Shank3^+/ΔC^ mice treated with saline or Rom/GSK (0.25/5 mg/kg, i.p., 3x) at 8 days post-treatment (*n* = 10–12 cells per group). Inset: representative NMDAR-EPSC traces. In all figures, ^#^*p* < 0.1, *p < 0.05, ***p < 0.001.

Finally, we examined whether treatment of adult Shank3^+/ΔC^ mice with Romidepsin and GSK-LSD1 may reverse synaptic dysfunction in PFC. Whole-cell patch-clamp recording was performed to measure spontaneous excitatory postsynaptic current (sEPSC) and evoked AMPAR- or NMDAR-mediated EPSC in layer V PFC pyramidal neurons. The amplitude and frequency of sEPSC had no difference between Shank3^+/ΔC^ and WT mice (Fig. 5A, B, *n* = 20 cells/3–4 mice/group, *p* > 0.05, *t*-test). NMDAR-EPSC to AMPAR-EPSC ratio was significantly reduced in PFC neurons of Shank3^+/ΔC^ mice (Fig. 5C, *n* = 15 cells/3–4 mice/group, *p* = 0.0003, t test), while the amplitude of AMPAR-EPSC evoked by a series of stimulus pulses were unchanged in PFC neurons of Shank3^+/ΔC^ mice (Fig. 5D, *n* = 13–15 cells/3–4 mice/group, *F*_4,104 (interaction)_ = 0.88, *p* > 0.05; *F*_1,26 (group)_ = 0.66, *p* > 0.05, two-way ANOVA). Thus, adult Shank3^+/ΔC^ mice have specific NMDAR hypofunction in PFC pyramidal neurons, similar to the pronounced phenotype found in young Shank3^+/ΔC^ mice^14,23,36^. Treatment of adult Shank3^+/ΔC^ mice with Romidepsin and GSK-LSD1 significantly increased the amplitudes of evoked NMDAR-EPSC in PFC neurons, compared to saline-treated Shank3^+/ΔC^ mice (Fig. 5E, *n* = 10–12 cells/3 mice/group, *F*_8,120 (interaction)_ = 8.2, *p* < 0.0001; *F*_2,30 (group)_ = 5.0, *p* = 0.01, two-way ANOVA). Collectively, these findings indicate that combined inhibition of HDAC and LSD1 restores the synaptic expression of NMDA receptor subunits, producing a robust recovery of NMDAR function in PFC of adult Shank3^+/ΔC^ mice.

## Discussion

To date, there is little information on the treatment strategy for adult ASD. Here, we show that combined pharmacological inhibition of epigenetic enzymes, class I HDAC and LSD1, is capable of reversing social deficits and hyper-aggressive behavior in adult male Shank3^+/ΔC^ mice. The dual inhibitors increase H3K9 acetylation and H3K4 dimethylation in PFC of adult Shank3^+/ΔC^ mice, resulting in transcriptional upregulation of NMDAR subunits, which is coupled with the elevated synaptic expression of NMDAR subunits and the restored NMDAR function in PFC pyramidal neurons. These findings indicate that the epigenetic and synaptic routes that drive behavioral improvements in Shank3^+/ΔC^ mice remain modifiable through adulthood.

When administered individually, the HDAC inhibitor Romidepsin and the LSD1 inhibitor GSK-LSD1 each confer modest and short-lived benefits on sociability in adult Shank3^+/ΔC^ mice. In contrast, combined treatment with dual inhibitors induces persistent rescue (up to 24 days) of social preference in adult Shank3^+/ΔC^ mice. We hypothesize that the synergistic effect of Rom/GSK is driven by the combined inhibition of two functionally coupled epigenetic enzymes, as class I HDAC and LSD1 have been shown to operate jointly in regulating gene transcription^19^. Indeed, compounds inhibiting both HDAC and LSD1 have synergistic actions in regulating apoptosis^37,38^, and combined HDAC/LSD1 inhibition has been proposed as a pharmacological approach for cancers that are unresponsive to single drug therapies^38^. In line with this idea, it appears likely that combined HDAC/LSD1 inhibition is necessary for persistent rescue of sociability in adult Shank3^+/ΔC^ mice.

Adult male Shank3^+/ΔC^ mice exhibit increased aggressive behavior, a new phenotype not found in young (juvenile to adolescent) Shank3^+/ΔC^ mice. Aggressive behavior is relatively common in individuals with ASD^39,40^. Aggression is also reported as one of the core symptoms in some humans with *Shank3* deletion (22q13.3 deletion syndrome)^41^. The prefrontal cortex has been highly implicated in aggressive behavior in both human and mice studies^42–45^. The alleviation of aggression in adult male Shank3^+/ΔC^ mice by combined HDAC/LSD1 inhibition indicates that this pharmacological intervention can improve several domains of behavioral abnormalities.

Combined administration of Romidepsin/GSK-LSD1 selectively increased expression levels of the NMDAR subunit genes *Grin2a/b*, while AMPAR subunit genes *Gria1/2* were unchanged. The exact mechanism for these differential transcriptional changes is unclear, though it may be driven by transcriptional control of selective gene targets by epigenetic enzymes. Chromatin modifications have been implicated in the regulation of developmental and experience-dependent processes in neurons, including synaptic plasticity^46^. We speculate that pharmacological inhibition of HDACs and LSD1 via Rom/GSK administration affects an epigenetic program specifically regulating NMDAR subunit expression in neurons, rather than AMPAR subunits or other genes related to synaptic plasticity. This transcriptional program may be experience-dependent rather than developmentally-controlled, as it remains treatment-responsive in PFC of adult mice.

*Shank3* encodes a scaffolding protein at glutamatergic synapses^8^, and *Shank3*-deficient mice exhibit NMDAR synaptic function deficits^14,47,48^. Restoring NMDAR activity in PFC – specifically via targeting epigenetic histone modifiers - is sufficient in rescuing sociability in adolescent Shank3^+/ΔC^ mice^13,14^. Here, we show that NMDAR hypofunction and reduced synaptic NMDAR expression, two phenotypes previously observed in adolescent Shank3^+/ΔC^ mice^13,14^, persist into adulthood, and these NMDAR deficits are reversed by Rom/GSK treatment in adult male Shank3^+/ΔC^ mice. We also observed that synaptic levels of NMDA receptor subunits were selectively restored in Romidepsin/GSK-LSD1-treated Shank3^+/ΔC^ mice, whereas total protein levels were unchanged by the treatment. It is unlikely that this is related to restored *Shank3* expression at synapses, as Rom/GSK treatment did not impact *Shank3* expression. We thus speculate that the restoration of synaptic NMDARs is driven by the increased receptor trafficking to synapses. Our previous studies have demonstrated that diminished synaptic distribution of NMDARs in Shank3^+/ΔC^ mice is mechanistically linked to β-catenin^13^ and actin regulators^14^, and Romidepsin treatment in adolescent Shank3^+/ΔC^ mice is capable of restoring sociability via rescuing actin filaments and synaptic NMDARs^13^. It is thus possible that Rom/GSK treatment is operating on similar molecular mechanisms in adult Shank3^+/ΔC^ mice.

Cumulatively, these results strongly suggest that therapies that can normalize glutamatergic transmission may offer high therapeutic potential for human *Shank3*-deletion carriers. Furthermore, this study shows that glutamatergic synaptic deficits in Shank3^+/ΔC^ mice remain pharmacologically amenable in adulthood. The restoration of PFC NMDAR function by combined inhibition of HDAC and LSD1 may underlie the behavioral effects of the dual inhibitors in adult Shank3^+/ΔC^ mice.
